# Probabilistic Solar Wind Forecasting Using Large Ensembles of Near‐Sun Conditions With a Simple One‐Dimensional “Upwind” Scheme

**DOI:** 10.1002/2017SW001679

**Published:** 2017-11-06

**Authors:** Mathew J. Owens, Pete Riley

**Affiliations:** ^1^ Space and Atmospheric Electricity Group, Department of Meteorology University of Reading Reading UK; ^2^ Predictive Science Inc San Diego CA USA

**Keywords:** solar wind, forecasting, ensembles, MHD

## Abstract

Long lead‐time space‐weather forecasting requires accurate prediction of the near‐Earth solar wind. The current state of the art uses a coronal model to extrapolate the observed photospheric magnetic field to the upper corona, where it is related to solar wind speed through empirical relations. These near‐Sun solar wind and magnetic field conditions provide the inner boundary condition to three‐dimensional numerical magnetohydrodynamic (MHD) models of the heliosphere out to 1 AU. This physics‐based approach can capture dynamic processes within the solar wind, which affect the resulting conditions in near‐Earth space. However, this deterministic approach lacks a quantification of forecast uncertainty. Here we describe a complementary method to exploit the near‐Sun solar wind information produced by coronal models and provide a quantitative estimate of forecast uncertainty. By sampling the near‐Sun solar wind speed at a range of latitudes about the sub‐Earth point, we produce a large ensemble (N = 576) of time series at the base of the Sun‐Earth line. Propagating these conditions to Earth by a three‐dimensional MHD model would be computationally prohibitive; thus, a computationally efficient one‐dimensional “upwind” scheme is used. The variance in the resulting near‐Earth solar wind speed ensemble is shown to provide an accurate measure of the forecast uncertainty. Applying this technique over 1996–2016, the upwind ensemble is found to provide a more “actionable” forecast than a single deterministic forecast; potential economic value is increased for all operational scenarios, but particularly when false alarms are important (i.e., where the cost of taking mitigating action is relatively large).

## Key Points

- An ensemble sampling of near‐Sun conditions provides a better solar wind forecast than a single deterministic forecast
- The ensemble approach both improves the “best” estimate and provides a reliable measure of solar wind forecast uncertainty
- The ensemble approach is particularly useful in operational settings where false alarms are important (i.e., high cost of mitigating action)

## 1 Introduction

Variability in near‐Earth solar wind conditions can lead to the energization of the terrestrial magnetosphere, resulting in disruption to power grids, communications, and satellite operations, as well as threat to health of humans in space and on high‐altitude aircraft (Cannon et al., 2013; Hapgood, 2011). Advanced forecasting of space weather is therefore highly desirable. State‐of‐the‐art forecasting is based on a coupled chain of specialized numerical models (e.g., see Figure 1 of Owens et al., 2014). The biggest challenge for long lead‐time (i.e., > day) space‐weather forecasting is accurate prediction of the near‐Earth solar wind conditions. Assuming the solar wind and magnetic field properties are known in the outer corona (typically 20 to 30 solar radii), mapping them to near‐Earth space is, in comparison, relatively straight forward: Numerical magnetohydrodynamic (MHD) models of the solar wind (e.g., Odstrcil et al., 2004; Riley, Linker, & Mikic, 2001; Tóth et al., 2005) are able to accurately represent large‐scale solar wind dynamics between the Sun and Earth (Odstrcil, 2003). But, ultimately, the ability to forecast the near‐Earth solar wind can only ever be as good as the near‐Sun boundary conditions.

Accurate determination of the near‐Sun solar wind conditions is difficult for a number of reasons. The most widely used method for determining synoptic near‐Sun solar wind properties is based on the reconstruction of the global coronal magnetic field via extrapolation of the observed photospheric magnetic field, using either the potential‐field source‐surface approximation (PFSS) (e.g., Mackay & Yeates, 2012, and references therein), or a global MHD model of the corona (Linker et al., 1999; Mikic et al., 1999). As observations of the photospheric magnetic field are only made from Earth or near‐Earth space, complete longitudinal coverage is achieved through solar rotation, and thus, it is necessary to assume quasi‐steady state conditions over the 27 day period. Similarly, observations limited to the Sun‐Earth line result in poorly reconstructed polar regions. Nevertheless, photospheric magnetic field extrapolation has been shown to provide good agreement with both eclipse observations (Mikic et al., 2007) and the polarity of the heliospheric magnetic field (HMF) (Owens & Forsyth, 2013), suggesting that the coronal magnetic field structure is reasonably well reproduced. The empirical relations between coronal magnetic field topology and solar wind speed, however, are relatively weak (McGregor et al., 2011; Riley, Linker, & Arge, 2015). Furthermore, and particularly at solar minimum, the position and width of the modeled slow wind band can be quite sensitive to the strength of the poorly observed polar photospheric fields, leading to large solar wind speed uncertainties at the sub‐Earth point (Bertello et al., 2014; Sun et al., 2011). Other observations, such as interplanetary scintillation (e.g., Breen et al., 2006) or heliospheric imagers (Eyles et al., 2009), can in principle provide a more direct measure of near‐Sun solar wind conditions. But using such data to provide the synoptic inner boundary conditions to solar wind models is complicated by line‐of‐sight effects, limited spatial coverage of observations, and lack of any HMF information.

This issue of uncertainty in solar wind forecasts has been investigated using relatively small (*N* = 10) ensembles of PFSS and MHD coronal models constrained with photospheric magnetic field observations from different observatories (Linker et al., 2017; Riley et al., 2013; Riley, Linker, & Mikić, 2013). For these initial proof‐of‐concept studies, only a single Carrington rotation was considered. The near‐Earth solar wind was found to have high sensitivity to the photospheric boundary conditions. For the limited period considered, the ensemble approach did provide a more reliable forecast than any individual realization, suggesting that the approach is worth pursuing.

Here we describe a complementary solar wind forecasting technique. It is based on the idea that photospheric magnetic field extrapolation techniques, such as PFSS and coronal MHD models, accurately reproduce the general solar wind speed structure at the top of the corona. However, (unknown) positional errors, particularly in the location of the slow solar wind band, mean that a single deterministic forecast of the near‐Earth solar wind speed does not fully exploit the near‐Sun solar wind information. Instead, we sample the near‐Sun solar wind conditions at a large range of positions around the sub‐Earth point and map them to near‐Earth space using a computationally efficient “upwind” scheme. This large ensemble of near‐Earth solar wind speeds is used to construct a probabilistic solar wind forecast with a quantitative estimate of the forecast uncertainty. The forecast value of this approach is compared with that from a single deterministic MHD forecast and climatology.

## 2 Data and Methods

To demonstrate the principle of using a large ensemble of near‐Sun solar wind conditions to make a probabilistic forecast of the near‐Earth conditions, we use the output of the “Magnetohydrodynamics Around a Sphere” (MAS) (Linker et al., 1999; Riley et al., 2012) global coronal model. However, we expect the results shown here to be generally applicable to any method of estimating the near‐Sun solar wind speed. MAS is constrained by photospheric magnetic field observations, which are extrapolated outward to 30 solar radii (*r_S_*), while self‐consistently solving the plasma and magnetic field parameters on a nonuniform grid in polar coordinates, using the MHD equations and the vector potential **A** (where the magnetic field, **B**, is given by ∇ × **A**), such that ∇.∇ × **A** = 0 (which ensures that current continuity, ∇. **J** = 0, is conserved to within the model's numerical accuracy). The MAS solutions used in this study are available from http://www.predsci.com/mhdweb/home.php.

Throughout this study, we use ambient MAS solutions relying on Carrington maps of the photospheric magnetic field. Thus, at the “forecast” time (although all results here are, strictly speaking, a “hindcast”), the data at the sub‐Earth point vary in “age” between 0 and 27 days, depending on the Carrington longitude. Clearly, this is not ideal for genuine forecast purposes; for operational forecasting, daily updated photospheric maps are generally used, wherein the data at the sub‐Earth point are always the most recent observation (of course, there must still be approximately 27 day old data in the photospheric map directly adjacent to the most recent observations, as longitudinal sampling is provided only by solar rotation). Thus, when we compare model results with observed solar wind conditions, we are not aiming to quantify the real forecast capability of the approaches discussed below. But by comparing relative skills of different techniques, we can establish whether the method proposed here has value for forecasting.

**Figure 1:**
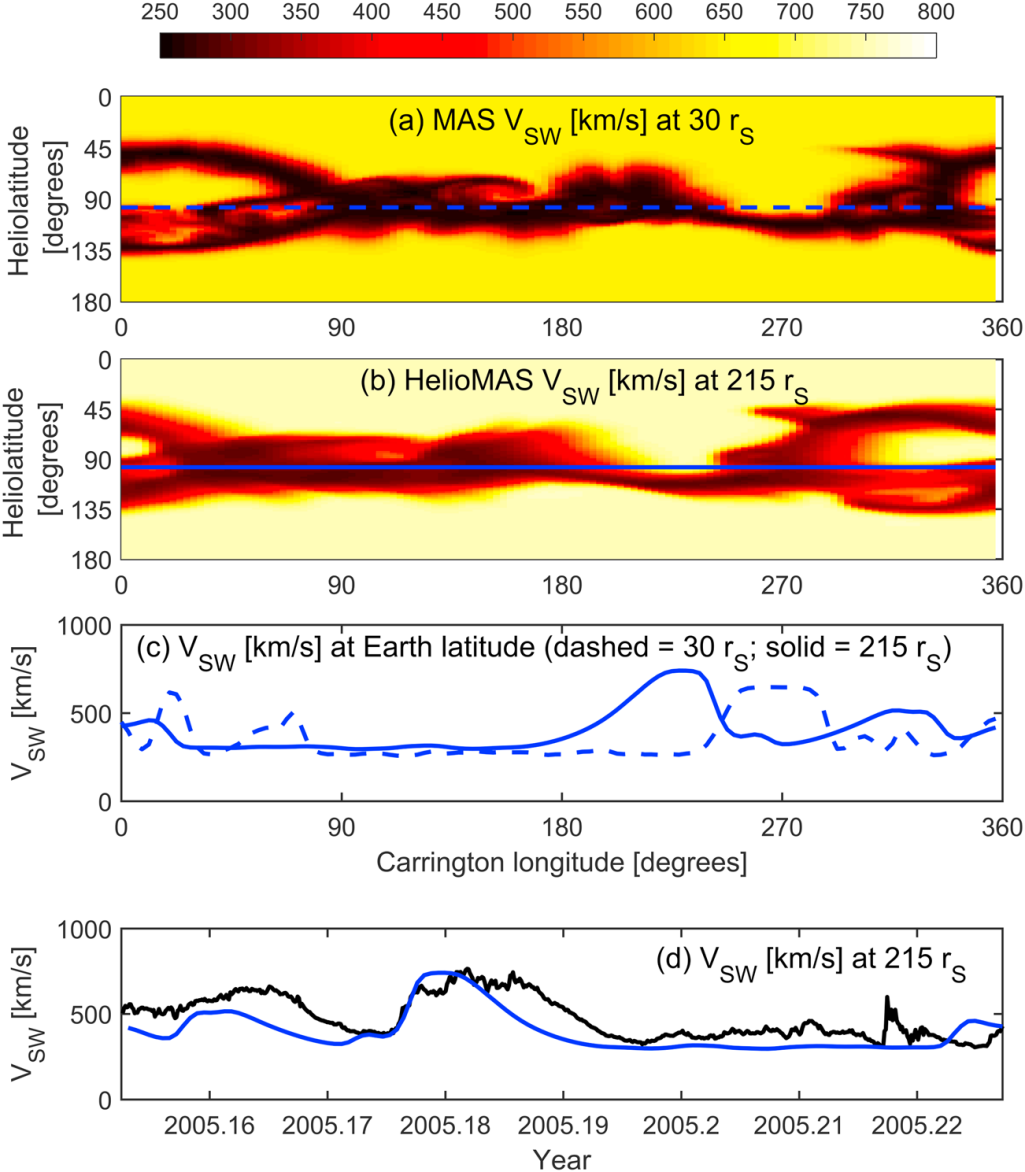
MAS and HelioMAS solution to the observed photospheric magnetic field for Carrington rotation 2027 (approximately spanning March 2005). (a) A Carrington longitude‐heliolatitude map of solar wind speed at 30 solar radii (*r_S_*) from MAS. The dashed blue line shows the sub‐Earth point as a function of Carrington longitude. (b) Resulting solar wind speed at 215 *r_S_* (1 AU) from the HelioMAS model. The solid blue line shows Earth orbit. (c) Solar wind speed at Earth latitude at 30 *r_S_* (blue dashed line), which has been propagated to 215 *r_S_* using the full 3‐D HelioMAS MHD model (blue solid line). (d) Same as Figure 1c but shown as a function of time, with OMNI observations shown in black.

Figure 1 shows a typical MAS solution, using the observed photospheric magnetic field for Carrington rotation (CR) 2027, which approximately spans March 2005. Figure 1a shows the solar wind speed at 30 *r_S_*, the outer boundary to the coronal solution. Earth orbit is shown as a dashed blue line, just below the heliographic equator at this time. The solar wind speed at the sub‐Earth point is shown as the dashed blue line in Figure 1c. The solar wind speed at 30 *r_S_* (and magnetic field, density, and temperature) is projected out to 1 AU (=215 *r_S_*) using the heliospheric version of MAS (here referred to as HelioMAS; Riley et al., 2001), also a full 3‐D MHD code, driven by boundary conditions based on the MAS coronal solution (Riley, 2010). The Enlil model (Odstrcil, 2003) is also widely used for such purposes. The HelioMAS solar wind speed at 1 AU is shown in Figure 1b, with Earth orbit shown as the solid blue line and the solar wind speed at this location shown as the solid blue line in Figure 1c. There is an approximately 50° shift in Carrington longitude between solar wind features at 30 *r_S_* and 1 AU, corresponding to the solar synodic rotation that occurs during the solar wind transit time (~3–4 days). Figure 1d displays solar wind speed at 1 AU as a function of time, rather than Carrington longitude, with the observed values shown as a solid black line. For this CR, there is reasonable agreement between the MHD solution and observations: The high‐speed stream midway through the CR is well predicted, although it is observed to last longer than the forecast. A smaller speed enhancement near the beginning of the interval is not very well captured by the MHD solution.

The full 3‐D MHD approach of HelioMAS reproduces the dynamical physics of the solar wind and HMF (Riley et al., 2001), and thus captures processes such as compression and rarefaction, solar wind stream deflection, HMF draping and magnetic field draping, and reconnection. It is, however, fairly computationally expensive, requiring several CPU hours per Carrington rotation at the resolution shown here (141 by 128 by 111 grid cells in the radial, longitudinal, and latitudinal directions, respectively). Thus, running an ensemble of solar wind solutions for different initial conditions would be limited to perhaps 10^2^ ensemble members. In order to efficiently explore the effect of uncertainty in the near‐Sun solar wind speed, we use a simple 1‐D upwind method of projecting solar wind speeds between 30 *r_S_* to 1 AU (Riley & Lionello, 2011), outlined below.

## 3 The One‐Dimensional Upwind Solar Wind Propagation Tool

By assuming time‐stationary solar wind flows (which is also an assumption in the full 3‐D MHD solar wind models, in the absence of time‐dependent boundary conditions), and neglecting the effects of magnetic field, gravity, and pressure gradients, Riley and Lionello (2011) showed that the solar wind on a discretized grid can be represented as

$$v_{r+1,\emptyset }=v_{r,\emptyset }+\frac{\Delta r\;\Omega_{\mathrm{ROT}}}{v_{r,\emptyset }}\;\left[\frac{v_{r,\emptyset +1}-v_{r,\emptyset }}{\Delta \emptyset }\right]$$

where subscripts *r* and *φ* are indices of radial and longitudinal grid cells, respectively; Δr and Δ*φ* are radial and longitudinal cell spacings, respectively; and Ω_ROT_ is the solar rotation frequency. Here we match the MAS longitudinal grid resolution by setting Δ*φ* = 2.18°, and set Δ*r* = 1 *r_S_*. The solutions obtained are essentially identical when the grid resolutions are increased by an order of magnitude, suggesting the Courant‐Friedrichs‐Lewy condition necessary for numerical convergence is well met.

**Figure 2:**
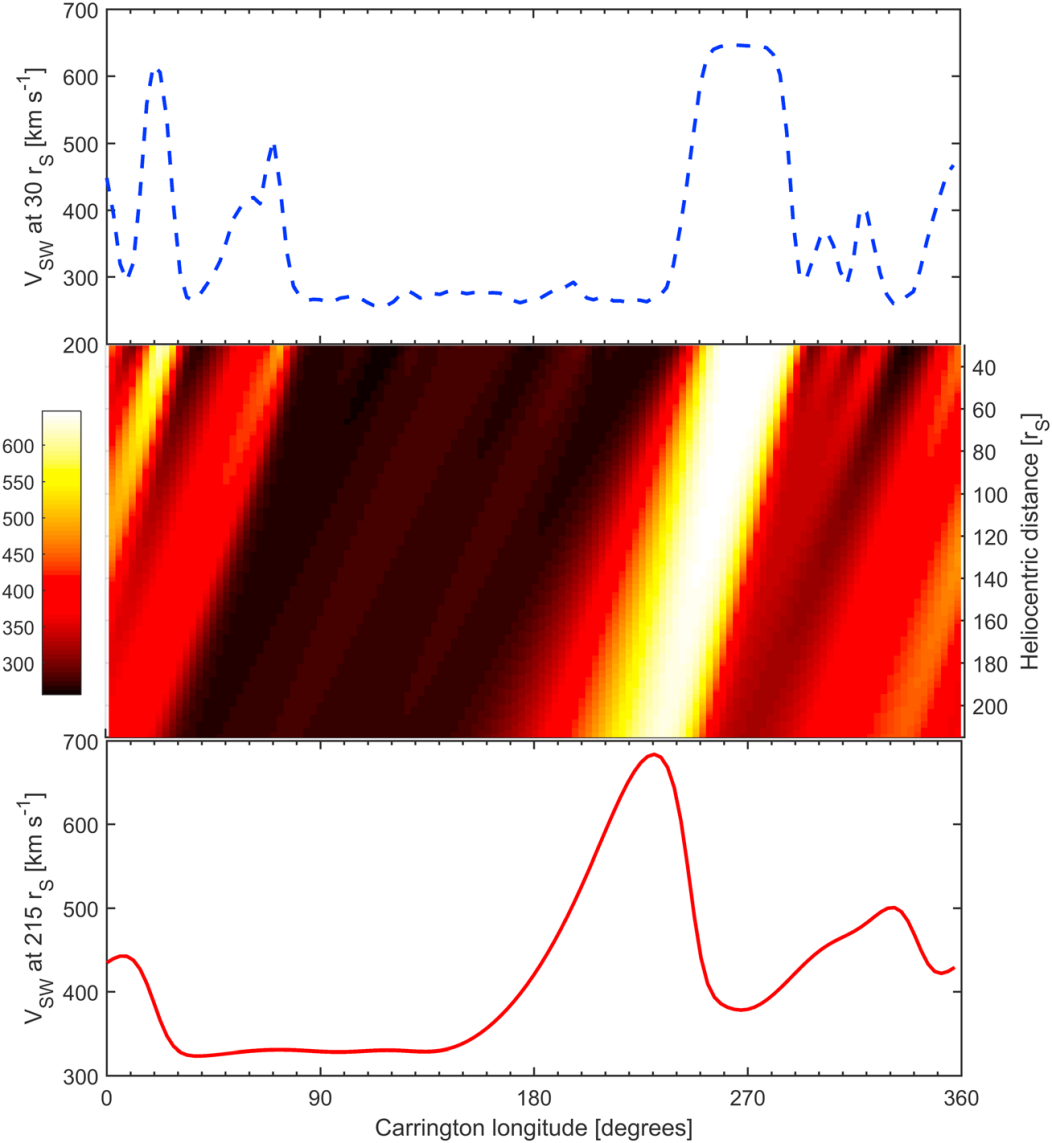
An example of the 1‐D upwind solution to solar wind speed between 30 and 215 r_S_. (top) The input solar wind speed at 30 *r_S_* from the MAS solution to the observed photospheric magnetic field for Carrington rotation 2027. (middle) Solar wind speed as a function of heliocentric distance and longitude from the upwind solution. (bottom) The resulting solar wind speed at 1 AU (215 *r_S_*).

To account for the residual solar wind acceleration between 30 *r_S_* and 215 *r_S_*, we also follow Riley and Lionello (2011) and include an additional speed contribution, *v*_ACC_ (*r*):

$$v_{\mathrm{ACC}}=\alpha v_{0}\;\left[1-e^{-r/r_{H}}\right]$$

where *v*_0_ is the initial solar wind speed (at 30 *r_S_*), *r* is the heliocentric distance, and the constants *α* and *r_H_* are set to 0.15 and 50 *r_S_*, respectively, which were found to provide reasonable fits to the 1 AU speeds. Thus, *v*_ACC_ varies between approximately 40 and 100 km s^−1^, depending on the initial solar wind speed. Figure 2 summarizes this upwind solution between 30 and 1 AU using as input the ecliptic solar wind speed at 30 *r_S_* from the MAS solution to CR2027 (the blue dashed line in both Figures 2a and 1c).

**Figure 3:**
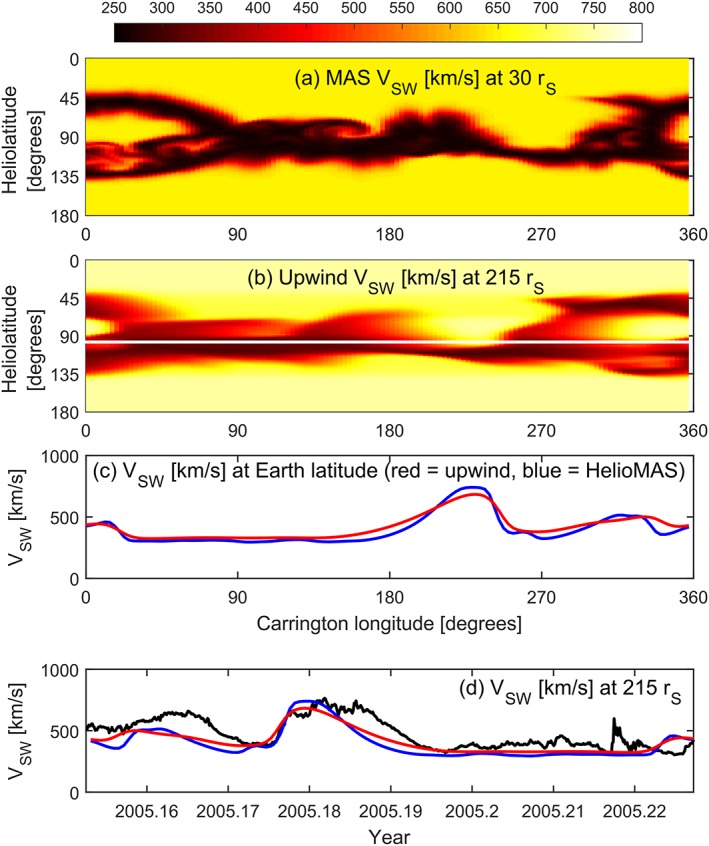
(a and b) MAS and upwind solution to the observed photospheric magnetic field for Carrington rotation 2027, in the same format as Figure 1. Figure 3b shows the upwind solution at 215 *r_S_* with Earth orbit as a white line. The red and blue lines show the (c) upwind and (d) HelioMAS solar wind speeds, respectively.

While the upwind method is essentially one dimensional, Figure 3b shows the individual solutions at 215 *r_S_* for all latitudes and longitudes. When compared to the three‐dimensional HelioMAS solution to the same 30 *r_S_* boundary condition (Figure 1b), it can be seen that the general structure is the same, but the solar wind speed contrast is reduced. The red lines in Figures 3c and 3d show the solar wind speed as a function of Carrington longitude and time, respectively, with the HelioMAS solution shown in blue. There is reasonable agreement, although the upwind solution is generally smoother, failing to quite meet the extremes of the 3‐D HelioMAS solution. The advantage of the 1‐D upwind method, however, is that it can be run on a modest desktop computer in a fraction of a second, allowing large ensembles of initial conditions to be investigated, as described below.

## 4 Producing a Solar Wind Speed Ensemble

**Figure 4:**
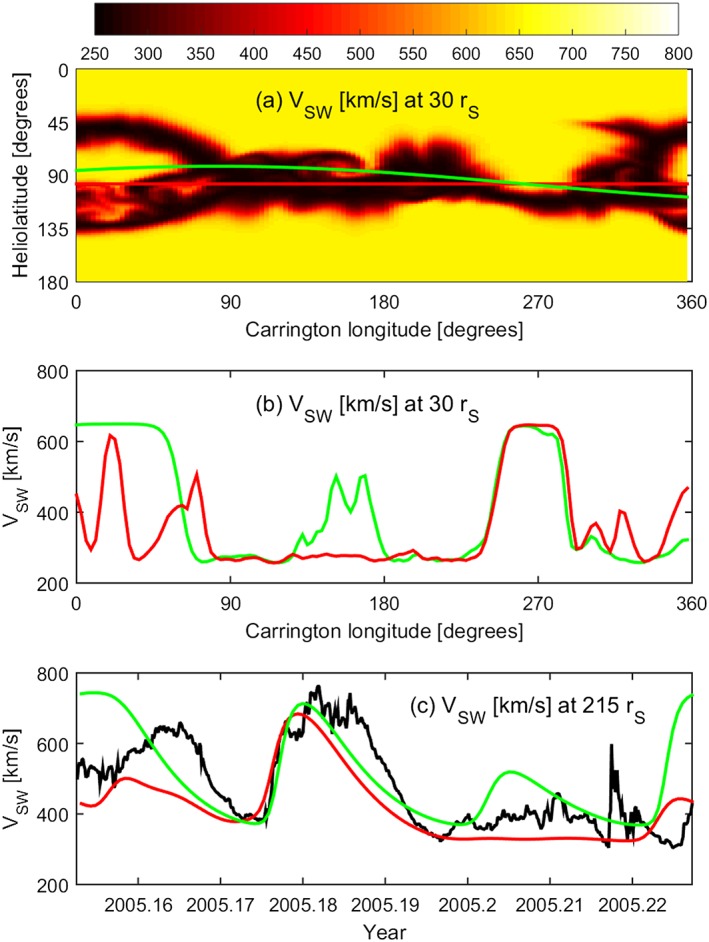
(a) Solar wind speed at 30 *r_S_* from the MAS solution to the observed photospheric magnetic field for Carrington rotation 2027 (approximately spanning March 2005). The red line shows Earth latitude; the green line shows a latitudinal perturbation about Earth latitude. (b) Solar wind speed at 30 *r_S_* at Earth latitude (red) and at the perturbed latitude (green), intended to mimic positional errors in the MAS solar wind speed solution. (c) Solar wind speed at 215 *r_S_* (=1 AU) using the upwind projection of the 30 *r_S_* solar wind speed at Earth latitude (red) and at the perturbed latitude (green). The black line shows the OMNI observations: This particular latitudinal perturbation does not provide an improvement in the near‐Earth solar wind speed.

With the necessary tools in place, the next step is to produce an ensemble of near‐Sun solar wind conditions to account for positional errors in the solar wind solution relative to the sub‐Earth point. This could be most directly achieved by spatially distorting the MAS solution and resampling at the sub‐Earth point, but this would be computationally expensive. The equivalent, but simpler, alternative taken here is to displace the path of sub‐Earth point relative to the solar wind solution. At each Carrington longitude (and therefore time step in the steady state approximation), we sample a range of latitudes about the sub‐Earth point. Truly random sampling within some latitudinal range is not desirable, as in practice, there is likely to be correlation between the latitudinal error at adjacent longitudes (though, of course, we cannot know what the latitudinal error actually is, or else an ensemble would not be required). To produce autocorrelated latitudinal deviations as a function of longitude, we adopt a simple approach. The perturbed latitude, *θ*, at which the solar wind is sampled for a given Carrington longitude, *φ*, is given by

$$\theta \left(\varphi \right)=\theta_{E}+\theta_{\mathrm{MAX}}\sin\left[n\;\varphi +\varphi_{0}\right]$$

where *θ_E_* is the (unperturbed) latitude of Earth, *θ*_MAX_ is the amplitude of the latitudinal deviation, *n* is the wave number of the latitudinal deviation that effectively determines the order of the “crinkling” of the MAS solar wind solution with respect to the sub‐Earth path, and *φ*_0_ is the longitudinal phase offset. The red line in Figure 4a shows Earth orbit, while the green line shows a perturbed latitudinal profile using *θ*_MAX_ = 15°, *n* = 0.5, and *φ*_0_ = 230°. The near‐Sun solar wind speeds at Earth and the perturbed latitude are shown in Figure 4b. The main difference is a large increase in fast wind at the perturbed latitude between Carrington longitudes of 0 and 90°. When used with the upwind model, this results in fast wind at 1 AU near the start and end of CR2027 (Figure 4c). As can be seen by comparison with the OMNI data shown in black, this particular perturbed latitudinal sampling of near‐Sun solar wind does not provide a significantly improved forecast of the observed near‐Sun solar wind speed. But by considering a large ensemble of latitudinal perturbations, a measure of the uncertainty in the solar wind forecast can be obtained, as is demonstrated below.

We produce an ensemble of solar wind speed profiles at 30 *r_S_* by sampling each MAS solution using *θ*_MAX_ = 0° to 15° in 1° steps, *n* = 0 to 1 in 0.5 steps, and φ_0_ = 0° to 330° in 30° steps (0° and 360° being identical). Thus, for each Carrington rotation, there are 576 individual *V*_SW_ profiles at 30 *r_S_*, of which Figure 4b shows just a single instance. The choice of bounds and step size for *n* and *φ*_0_ does not significantly affect the results presented here, and the values have been chosen simply to ensure that all latitudes are well sampled at each longitude. In principle, *θ*_MAX_ can have more of an effect. If the upper bound of *θ*_MAX_ is too low, the ensemble simply collapses to the unperturbed profile. If *θ*_MAX_ is too large, the full range of solar wind speeds is available at almost all longitudes, and thus, the forecast uncertainty is likely to be highly overestimated. We note, however, that using upper bounds on *θ*_MAX_ of 10, 15, and 20°, which are values comparable to the typical angular extent of the slow wind band (Owens, Crooker, & Lockwood, 2014), all produce qualitatively similar results.

**Figure 5:**
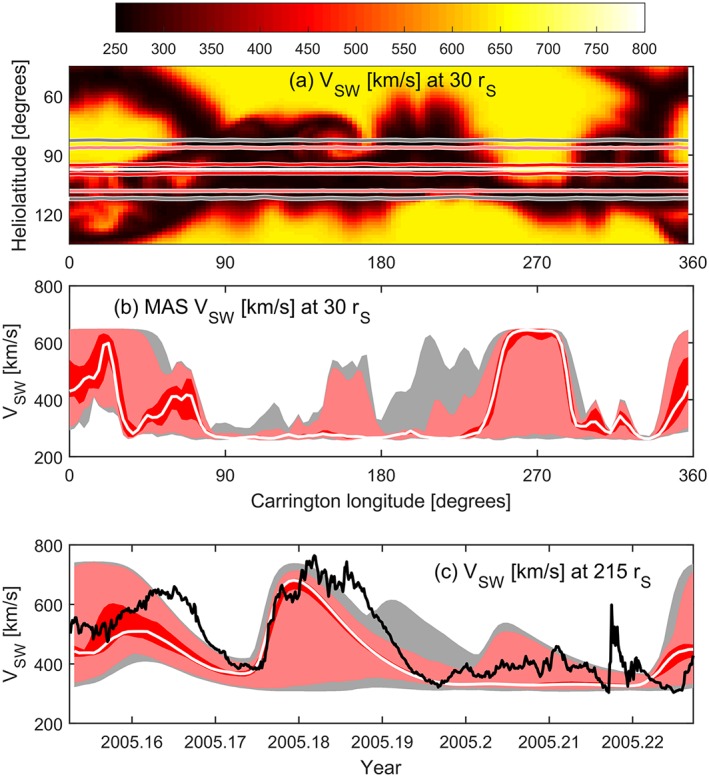
The upwind ensemble method applied to Carrington rotation 2027. (a) Solar wind speed at 30 *r_S_* from the MAS solution to the observed photospheric magnetic field. Note the reduced latitudinal range of the plot compared to previous figures. The red, pink, and grey lines show the latitudinal ranges containing 68%, 95%, and 99.7% (i.e., 1‐sigma, 2‐sigma, and 3‐sigma quantiles) of the total ensemble samples, with the median latitude, Earth orbit, in white. (b) The resulting ensemble of near‐Sun solar wind speeds, in the same format. (c) The resulting upwind ensemble at 215 *r_S_*, in the same format. The observed solar wind speed is shown in black.

Each sampling of the MAS solar wind speed at 30 *r_S_* is then individually projected to 1 AU using the upwind scheme. Figure 5 shows the upwind ensemble method applied to CR2027. Figure 5a shows the MAS solution at 30 *r_S_* from which the inner boundary conditions to the upwind model are sampled. The red, pink, and grey lines show the latitudinal ranges, which contain 68, 95, and 99.7% (i.e., 1‐sigma, 2‐sigma, and 3‐sigma quantiles) of the samples in the ensemble. It can clearly be seen that this sampling is essentially uniform with longitude. From approximately 90° to 230° and 300° to 340° Carrington longitudes, the ensemble samples almost completely within slow wind band. At other longitudes, a mixture of fast and slow wind is sampled. The resulting ensemble of initial conditions is shown in Figure 5b and the near‐Earth conditions from the upwind ensemble in Figure 5c. When the ensemble median, shown in white, gives slow wind, there is generally low ensemble spread, whereas when the median gives fast wind, greater ensemble spread is present (as, at least for this CR, slow wind is always present too).

**Figure 6:**
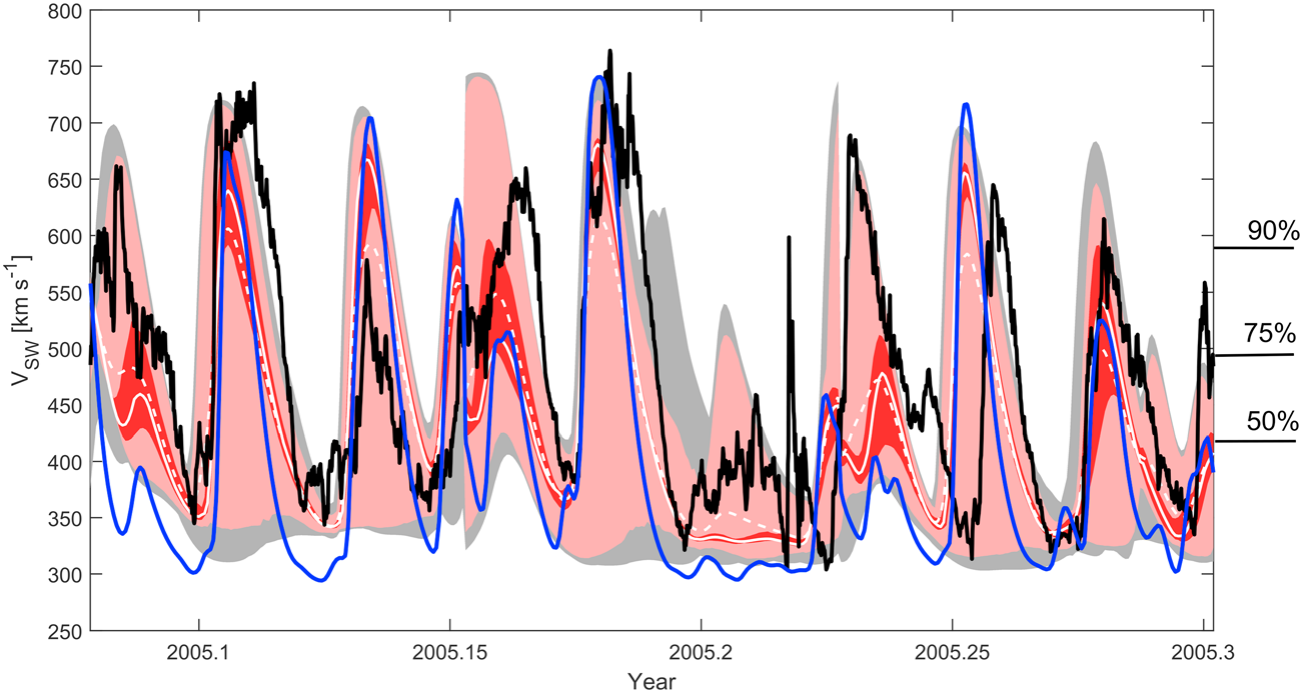
A time series of near‐Earth solar wind speed for Carrington rotations 2026 through 2028. The black line shows the observed solar wind speed from OMNI spacecraft. The blue line shows the solar wind speed obtained from the full 3‐D MHD HelioMAS solution. The dashed and solid white lines show the mean and median, respectively, of the 576‐member ensemble of MAS solar wind speeds at 30 *r_S_*, projected to 1 AU using the upwind model. The red, pink, and grey shaded regions show the 1‐sigma, 2‐sigma, and 3‐sigma quantiles in the upwind ensemble, respectively. The numbers on the right‐hand axis show the percentiles of the entire OMNI data set.

An example of the solar wind speed ensemble at 1 AU for a longer time period is shown in Figure 6, with the full 3‐D MHD solution (i.e., HelioMAS) also shown in blue for comparison. The mean and median of the upwind ensemble, shown as the dashed and solid white lines, respectively, are generally within 50 km s^−1^ of the single MHD solution. For this short period, the root‐mean‐square error (RMSE) of the single MHD solution is 134 km s^−1^, while the upwind ensemble median (mean) has an RMSE of 102 (92) km s^−1^, a modest but statistically significant improvement. The upwind ensemble median, rather than mean, is used as the “best” estimate, as the distribution of solar wind speeds at a given time step is generally not Gaussian, but highly skewed (thus, the ensemble mean often lies outside the 1‐sigma uncertainty band, such as at 2005.23). Thus, the ensemble mean tends to further reduce the dynamic range of solar wind speed forecast, and while it results in a lower RMSE, it may not necessarily provide a more “useful” forecast for a space‐weather operator.

Of course, the primary purpose of quasi‐random perturbation of the initial conditions is not necessarily to provide a systematic improvement of the “most probable” forecast. Instead, we anticipate that this approach will provide an accurate assessment of the forecast uncertainty, by measuring the sensitivity to small latitudinal errors in the near‐Sun solar wind speed. As can be seen by the red, pink, and grey shaded areas in Figure 6, the 1‐sigma, 2‐sigma, and 3‐sigma quantiles of the upwind ensemble can deviate substantially from ensemble median. At the 1‐sigma quantile, the probable range of solar wind speeds remains quite narrow. At 2‐sigma and 3‐sigma quantiles, however, the range of predicted solar wind speeds is broad during times when the ensemble median forecasts high solar wind speeds, though remains narrow during times when the median is at low speeds. Note also that when the ensemble spread is largest, it is generally highly asymmetric with high wind speeds being more probable than low wind speeds. At least for this specific interval, there is low ensemble spread (interpreted as high confidence in the solar wind speed forecast) whenever slow wind is forecast (by either the ensemble median or the single MHD solution). There is one exception around 2005.20–2005.22, where the ensemble median suggests very slow wind, but the ensemble spread suggests a possibility of intermediate speed wind, as is observed. To determine how accurately the ensemble spread is representing the forecast uncertainty requires more complex metrics than simple RMSE.

## 5 Cost/Loss Analysis

While these results seem generally promising, it is necessary to quantify whether a probabilistic forecast with relatively large uncertainties (i.e., the upwind ensemble) adds forecast value above the single deterministic forecast (i.e., HelioMAS, in this case). Of course, the answer to this question will depend on the operational setting in which the forecasts are to be used. We adopt the “cost/loss” analysis developed for meteorological applications (Murphy, 1985), as it both takes into account different forecast requirements and allows direct comparison of deterministic and probabilistic forecasts. It indirectly allows us to assess whether the probabilities of the solar wind speed exceeding certain thresholds are an accurate measure of the forecast uncertainty. This approach was applied to space weather forecasting by Owens et al. (2014) and Owens, Riley, and Horbury (2017), but is summarized again here.

**Figure 7:**
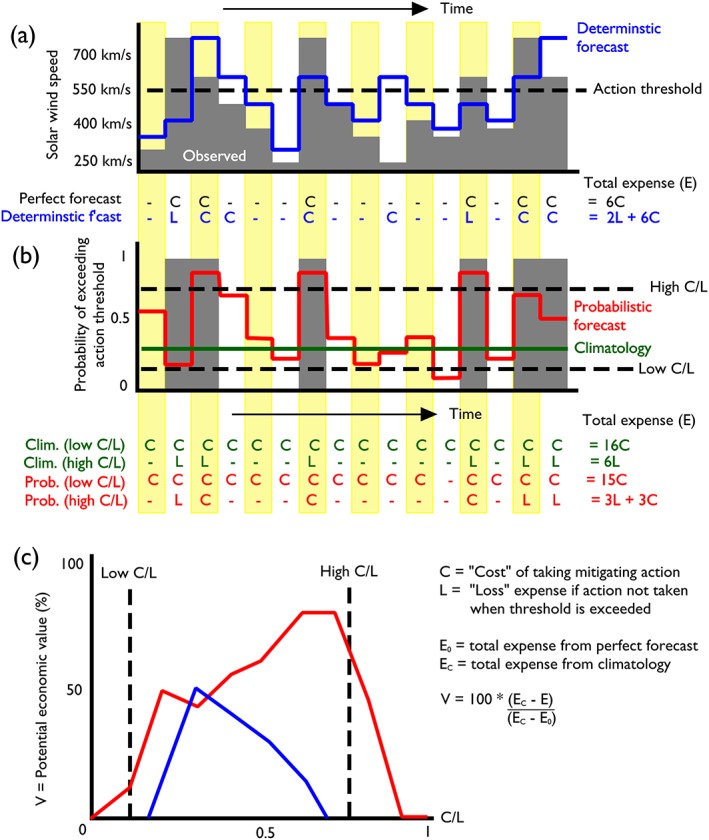
A summary of “cost/loss” analysis. (a) Sketches of solar wind time series, with grey shading indicating observed solar wind, the blue line showing a deterministic forecast, and the black dashed line showing the threshold at which an operator would take mitigating action. The cost of taking mitigating action is C, while the loss expense of not taking action when the threshold is exceeded is L. The total expense, *E*, of acting on any forecast is the sum of the total C and L incurred, shown below Figure 7a. (b) The probability of exceeding the action threshold. Observations are grey, the red line shows a probabilistic forecast, and the green line shows the climatological probability. (c) The potential economic value of the deterministic (blue) and probabilistic (red) forecasts relative to climatology and a perfect forecast, for the range of possible C*/*L values. See text for further explanation.

Figure 7a shows a cartoon of solar wind speed time series. Let us assume that a forecaster or operator will take mitigating action, such as putting the spacecraft or power system into safe mode, if the solar wind speed exceeds some threshold. In the example shown, a threshold of 550 km s^−1^ has been chosen, which is exceeded at 6 of the 16 time steps. Thus, if the operator was able to act on the basis of a perfect deterministic forecast, a total expense (*E*_0_) of 6C would be incurred, where C is the cost of taking mitigating action, for example, from lost revenue. Now consider the illustrative deterministic forecast, shown in blue. It predicts that the solar wind speed will exceed the action threshold for 6 of the 16 intervals, incurring an expense of 6C, but it also misses two periods when the action threshold is exceeded, so the total expense is *E* = 2L + 6C, where L is the loss expense for not taking action when it was required. Thus, while the total expense of a perfect and deterministic forecast varies with the value of C/L, as shown by Table 1, the decision about whether or not to take action on the basis of the forecast is independent of C/L.

**Table 1:** A Summary of the Total Expense for the Forecasts Illustrated in Figure 7, Over a Range of Cost/Loss (C/L) Values

C/L	Perfect forecast		Climatology		Deterministic forecast			Probabilistic forecast		
	Total expense, *E*_0_		Total expense, *E*_C_		Total expense, *E*		*V*	Total expense, *E*		*V*
0.01	6C	0.06	16C	0.16	2L + 6C	2.06	−1900	16C	0.16	0
0.10	6C	0.60	16C	1.60	2L + 6C	2.60	−100	15C	1.50	10
0.20	6C	1.20	16C	3.20	2L + 6C	3.20	0	11C	2.20	50
0.30	6C	1.80	6L	6.00	2L + 6C	3.80	52	10C + L	4.00	48
0.40	6C	2.40	6L	6.00	2L + 6C	4.40	44	7C + L	3.80	61
0.50	6C	3.00	6L	6.00	2L + 6C	5.00	33	6C + L	4.00	67
0.60	6C	3.60	6L	6.00	2L + 6C	5.60	17	5C + L	4.00	83
0.70	6C	4.20	6L	6.00	2L + 6C	6.20	−11	5C + L	4.50	83
0.80	6C	4.80	6L	6.00	2L + 6C	6.80	−67	3C + 3 L	5.40	50
0.90	6C	5.40	6L	6.00	2L + 6C	7.40	−233	6 L	6.00	0
0.99	6C	5.94	6L	6.00	2L + 6C	7.94	−3233	6 L	6.00	0

*Note*. Numbers in red show the values obtained for L = 1, while numbers in blue show the potential economic value, *V*, of the forecasts relative to a perfect forecast and climatology.

Figure 7b shows the probability of exceeding the 550 km s^−1^ solar wind speed threshold; thus, the observational value is either 0 or 1. The green line shows a climatological probability (*P*_C_, as would be determined from a much larger historical data set) of approximately *P*_C_ = 0.25. If the operator was acting solely on the basis of such a climatological forecast (i.e., that the probability of exceeding the action threshold is *P*_C_ at all times), the decision about whether or not to take action depends on the operational scenario. If taking action is cheap (i.e., C/L is low, say 0.1, as shown by the lower black dashed line), the operator should take action at all times and the total climatological expense, *E*_C_, is 16C. If, however, taking action is expensive (i.e., C/L is high, say 0.75, as shown by the upper black dashed line), the operator should never take action and *E*_C_ = 6L. The break between these two regimes is C/L = *P*_C_.

Table 1 shows the total expense for a perfect forecast, climatology, and the deterministic forecast over a range of cost/loss (C/L) values. The numbers in red show the values obtained for L = 1, though this choice is entirely arbitrary as L cancels out in the calculation of potential economic value, *V*. As shown by the equation to the right of Figure 7c, V is the ratio of difference in total expense between the forecast and climatology (i.e., *E* − *E*_C_) to the difference in total expense between a perfect forecast and climatology (i.e., *E*_C_ − *E*_0_), multiplied by 100 to convert to a percentage. *V* = 100% means the forecast is perfect, with no uncertainty in the forecast values, whereas *V* < 0% means the forecast performs worse than climatology. Thus, the deterministic forecast is better than or equal to climatology only in the range 0.2 ≤ C/L ≤ 0.6. At low C/L, the cost of false alarms is so low and missed events so high that a “cautious” forecast of always taking action is more sensible than using the imperfect deterministic forecast. At high C/L, missed events become less important and false alarms become costly, so never taking action is a more prudent course of action than using the imperfect deterministic forecast.

The red line in Figure 7b shows an illustration of a probabilistic forecast. Like climatology, the operator's decision of whether or not to take action will depend on C/L; when the probability of exceeding the *V*_SW_ threshold is greater than C/L, the operator should take action, whereas they should not when the forecast probability is less than C/L. Thus, for a low C/L of around 0.25, the total expense of the probabilistic forecast is *E* = L + 10C, whereas for a high C/L of around 0.75, *E* = 3L + 3C. Other values are shown in Table 1. As can be seen from Figure 7c, this results in *V* for the probabilistic forecast that is greater than or equal to zero for all C/L. At C/L = 0 and C/L = 1, *E* for the probabilistic forecast reduces to climatology (and *V* tends to 0), as there are no intervals with either 0% or 100% forecast probability of exceeding 550 km s^−1^. This highlights the fact that probabilistic forecasts are not intrinsically more valuable than deterministic ones, only when the stated probability accurately quantifies the forecast uncertainty does the probabilistic nature of the forecast add value. A probabilistic forecast that “hedges its bets” with roughly equal probability for all solar wind speeds will be penalized heavily at both high and low C/L ratios and thus provide a lower potential economic value. Such a forecast is not “actionable.”

**Figure 8:**
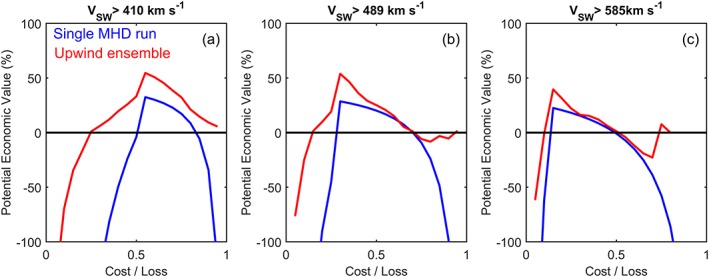
The potential economic value, relative to climatology, of a single deterministic forecast (HelioMAS, blue) and an upwind ensemble forecast (red) over a range of cost/loss values. Low cost/loss ratios represent operational scenarios in which missed events are most important, whereas high cost/loss ratios represent situations where false alarms must be minimized. Data shown are for Carrington rotations 2026–2028. Panels from left to right show increasing action thresholds (the 50th, 75th, and 90th percentiles of the observed solar wind, respectively).

Figure 8 shows the cost/loss analysis applied to the two solar wind speed forecast methods considered in this study (the deterministic MHD solution and the upwind ensemble method) for the three Carrington rotations shown in Figure 6. The panels, from left to right, show increasing *V*_SW_ thresholds at which a hypothetical operator takes action, chosen to be 410, 489, and 585 km s^−1^, the 50th, 75th, and 90th percentiles of *V*_SW_ over the whole OMNI data set (1963 to present). (These thresholds are shown on the right‐hand axis of Figure 6.) Potential economic value is relative to climatology, so values greater than 0 show regimes where the forecasts provide greater value than simply assuming a climatological probability of exceeding the *V*_SW_ action threshold at all times (i.e., 0.5 for 410 km s^−1^, 0.75 for 489 km s^−1^, and 0.9 for 585 km s^−1^). Normally, such potential economic value plots would be curtailed where the value drops below zero, but here the solar wind estimates are based on Carrington maps and thus are not being used in true “forecast mode.” Instead, we are interested in the relative value of the deterministic MHD and upwind ensemble forecast methods, rather than their absolute skill, and thus, negative values are still considered meaningful. For an action threshold at 410 km s^−1^ (i.e., a binary prediction of fast or slow wind, using the median as the discriminator), the upwind ensemble provides additional value at all C/L ratios, but particularly at low C/L, where missed events are important. For higher action thresholds (489 and 585 km s^−1^), the difference between the single MHD run and the upwind ensemble are less marked.

The increase in potential economic value for the probabilistic forecast relative over the deterministic forecast demonstrates that the ensemble spread is a good measure of forecast uncertainty, at least for this limited time period. The next section examines a much longer time interval.

## 6 Results for Interval 1996–2016

**Figure 9:**
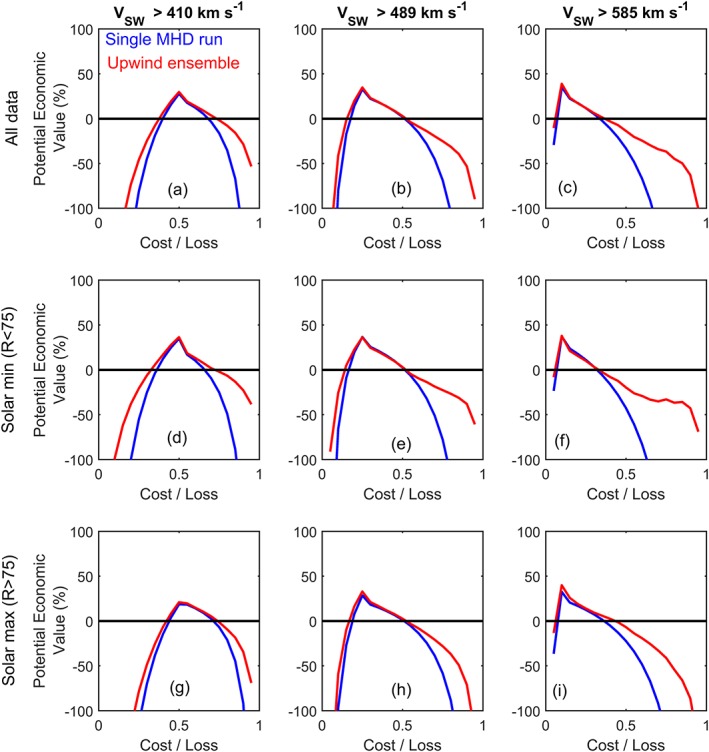
Same as Figure 8 but for the years 1996–2016 inclusive. (a–c) The whole data set and (d–f/g–i) solar minimum/maximum conditions, defined by a Carrington rotation average sunspot number below/above 75.

We now consider the usefulness of the upwind ensemble over a much longer interval: 1996–2016 inclusive, the period of near‐complete observational coverage in the near‐Earth solar wind. Over this extended period, we find the RMSE for the single MHD realization to be 123 km s^−1^, very similar to what has been reported by previous studies (Owens et al., 2008), while the upwind ensemble median (mean) has an RMSE of 107 (101) km s^−1^. Given the long time period considered, this increase in skill is statistically significant, but caution must always be taken in interpretation of a simple metric such as RMSE. As the upwind technique tends to smooth the 1 AU solar wind speed and does not capture the full solar wind speed extremes produced by the MHD solar wind model, it may produce a lower RMSE without providing a more actionable forecast; taken to an extreme, a purely climatological forecast of no solar wind variation can sometimes have a lower RMSE than an accurate solar wind forecast with small timing errors in a forecast. This occurs as the latter suffers from “double penalties” in RMSE, producing both a missed event and a false alarm, while climatology only gets penalized for the missed event (e.g., see Figure 4 of Owens et al., 2005). To better assess the usefulness of the upwind ensemble forecast, Figures 9a–9c show the potential economic value of the solar wind speed forecasts for a range of cost/loss ratios and action thresholds. The value of the upwind ensemble is equal to, or greater than, the single MHD run in all cases. The upwind ensemble provides particular gains at high C/L ratios, where a low false alarm rate is required. Figures 9d–9f and 9g–9i show the period split into solar minimum and maximum, respectively, using a Carrington rotation averaged sunspot number threshold of 75, which approximately bisects the time period. The high cost/loss improvement seems to be primarily a result of improved upwind ensemble forecast capability during solar minimum, as expected, though the difference between solar minimum and solar maximum is relatively small.

## 7 Discussion and Conclusions

This study has investigated a new means to exploit existing near‐Sun solar wind estimates from coronal models to provide a quantitative assessment of the uncertainty in near‐Earth solar wind forecasts. We assume that potential‐field source‐surface (PFSS) and numerical magnetohydrodynamic (MHD) model extrapolations of the observed photospheric magnetic field reproduce the general structure of the near‐Sun solar wind but are subject to small (unknown) positional errors. Thus, we sample the near‐Sun solar wind at a range of latitudes about the sub‐Earth point, effectively simulating a range of possible positional errors of the solar wind solution relative to the sub‐Erath point. This large ensemble (*N* = 576) of initial conditions is projected to 1 AU using a computationally efficient upwind scheme to produce a probabilistic estimate of the solar wind speed at 1 AU.

This upwind ensemble scheme was applied to the near‐Sun solar wind speed estimates from the MAS MHD coronal model using Carrington rotation maps of the photospheric magnetic field over the years 1996–2016 inclusive. Over this extended period, the best upwind ensemble forecast (i.e., the ensemble median) provided a lower root‐mean‐square error (RMSE) than the single deterministic MHD forecast. Thus, the upwind ensemble median is a better deterministic forecast, at least by the crude metric of RMSE. The real advantage of the ensemble approach, however, is that it provides a useful assessment of the forecast uncertainty. Using the probabilistic information to inform a potential economic value calculation, the upwind ensemble forecast is found to produce a more “useful” forecast of near‐Earth solar wind than the single deterministic forecast, over all forecast scenarios. The largest improvement is for operational scenarios in which false alarms are particularly important (i.e., when the cost of taking mitigating action is relatively high). Thus, the upwind ensemble technique provides a quick and computationally inexpensive forecast that is complementary to (deterministic) full three‐dimensional MHD solar wind solutions, as provided by HelioMAS or Enlil heliospheric codes, and can easily be adapted to existing deterministic solar wind forecast methods. Where small ensembles of coronal solutions are produced, for example, using different models, different photospheric magnetic field observations (Linker et al., 2017; Stevens et al., 2012), and/or different evolutions of the photospheric field (Hickmann et al., 2015), the upwind ensemble method could be easily applied independently to each coronal solution. The resulting “super ensemble” could then be combined, ideally using weightings based on past performance (Adhikari & Agrawal, 2012; Owens et al., 2016), to provide a more robust assessment of forecast uncertainty.

Of course, there are also a number of limitations to this general approach. In particular, here we have only applied it to solar wind speed, using the upwind method to project between 30 solar radii and 1 AU. In order to perform the same analysis for other solar wind parameters, the projection to 1 AU could be made with a simple one‐dimensional MHD model, which would still allow a relatively large ensemble of initial conditions to be considered. Alternatively, the uncertainty in solar wind speed measured by the upwind ensemble could be used as a general measure of uncertainty across all solar wind parameters. Both these approaches will be investigated in future studies.

It should also be noted that the ensemble method has here been applied to coronal solutions based on Carrington‐rotation maps of the photospheric field, in which the sub‐Earth observations are between 0 and 27 days old. In a true operational forecasting situation, daily updated photospheric maps would be used, so that the sub‐Earth photospheric observations are always the most recently available, somewhat reducing the forecast error introduced by the assumption that the Sun has not changed for 27 days. Daily updated maps could in principle result in a different form of the latitudinal uncertainty in the near‐Sun solar wind to Carrington maps and hence affect the ability of the upwind ensemble to determine the forecast uncertainty. In practice, however, the polar fields are poorly observed for both Carrington and daily updated photospheric maps, so similar latitudinal errors are expected.

Finally, the upwind ensemble only assesses the uncertainty in the quasi steady state solar wind and cannot be immediately applied to transient structures such as coronal mass ejections (CMEs). Ensembles based on a range of CME initial conditions have been used, though these ensembles are necessarily small (e.g., *N* = 30 to 40), as they require the full three‐dimensional MHD solar wind models, and can consequently only account for little‐to‐no uncertainty in the steady state solar wind into which the CMEs propagate (Cash et al., 2015; Mays et al., 2015). Effectively combining the benefits of upwind and CME ensembles will require a different approach, perhaps using simple quasi‐empirical “drag” models of CME propagation (Cargill, 2004; Vrsnak & Gopalswamy, 2002), which could be used with large ensembles of both CME and steady state solar wind conditions.
